# Authors’ Response to Peer Reviews of “Impact of COVID-19 Testing Strategies and Lockdowns on Disease Management Across Europe, South America, and the United States: Analysis Using Skew-Normal Distributions”

**DOI:** 10.2196/28893

**Published:** 2021-04-21

**Authors:** Stefano De Leo

**Affiliations:** 1 Department of Applied Mathematics State University of Campinas Campinas Brazil

**Keywords:** COVID-19, testing strategy, skew-normal distributions, lockdown, forecast, modeling, outbreak, infectious disease, prediction

*This is the author’s response to peer-review reports for "COVID-19 Testing Strategies and Lockdowns: The European Closed Curves, Analyzed by Skew-Normal Distributions, Forecasts for the United Kingdom, Sweden, and the United States, and the Ongoing Outbreak in Brazil."*

## Round 1 Review

The author of the manuscript [1] is grateful to the editor and reviewers [2,3] for their invaluable input and feedback.

### Response to Reviewer D

We thank the reviewer [2] very much for their positive report. As suggested, we have corrected the typos in the main text.

### Response to Anonymous

We thank the reviewer [3] for their comments. The main purpose of this paper was to prove that massive testing strategies are probably the best choice for managing the COVID-19 pandemic. This was clearly demonstrated in section II where the pandemic in Germany and Italy was analyzed. As observed by reviewer D, “the statistical analysis is quite enlightening, showing how the available data should be interpreted and used to improve health systems’ response to the crisis and explaining why the working strategies of countries like Germany and South Korea are so effective.” The mathematical points of this paper enabled us to predict the peak by a dynamical analysis, as shown in Figure 9, and the end of the outbreak by using skew-normal distributions. In our conclusions, we have added a discussion on these aspects to satisfy your suggestions.

## Round 2 Review

We thank the reviewer [3] for their suggestions and observations. Below we list responses and changes done in the revised version.

1. In the revised version, the table in section II, where we introduce the effectiveness factor (EF), is now labeled A and has a legend. The three tables in section III were reduced to two tables (B and C), and now appear with their corresponding legends. These tables are important to justify our discussion on the different testing strategies adopted by Italy, Germany, the United States, and Brazil, and show how the effective testing strategy of the German authorities made a great difference compared to Italy.
2. In the revised version of the manuscript, we, following the suggestions of the reviewer, added in some sentences on interpretation in the *Abstract* section: “The massive testing strategy adopted, in the early stage of the disease, by German authorities made a great difference with respect to other countries, in particular with respect to Italy, where an effective testing strategy was adopted too late. This explains why, despite a strictly indiscriminate lockdown, the mortality rate was one of the highest in the world.”
  The *Introduction* section of the revised version now begins with: “In this paper, by analyzing in detail the testing strategies of the German and Italian authorities, in the early stage of the COVID-19 disease, and fitting the pandemic curves by skew-normal distributions (this allows us to compare the outbreak spread in different European and American countries by mathematical parameters), we show how massive testing strategies are more effective than strictly containment measures (full lockdowns) adopted by some countries.”
3. Following the suggestions of the reviewer, in section III, we shortened our mathematical discussion.
