# Correction: Authors’ Response to Peer Reviews of “Impact of COVID-19 Testing Strategies and Lockdowns on Disease Management Across Europe, South America, and the United States: Analysis Using Skew-Normal Distributions”

**DOI:** 10.2196/29878

**Published:** 2021-04-26

**Authors:** Stefano De Leo

**Affiliations:** 1 Department of Applied Mathematics State University of Campinas Campinas Brazil

In “Authors’ Response to Peer Reviews of ‘Impact of COVID-19 Testing Strategies and Lockdowns on Disease Management Across Europe, South America, and the United States: Analysis Using Skew-Normal Distributions’” (JMIRx Med 2021;2(2):e28893) one error was noted.

The reviewer ID of the anonymous reviewer has been removed from the published article to preserve anonymity.

The correction will appear in the online version of the paper on the JMIR Publications website on April 26, 2021, together with the publication of this correction notice. Because this was made after submission to PubMed, PubMed Central, and other full-text repositories, the corrected article has also been resubmitted to those repositories.

